# Carthamin yellow attenuates brain injury in a neonatal rat model of ischemic–hypoxic encephalopathy by inhibiting neuronal ferroptosis in the hippocampus

**DOI:** 10.1515/tnsci-2022-0331

**Published:** 2023-12-31

**Authors:** Yuanyu Zhou, Yuebin Wang, Xiaoqing Wu, Junjie Wu, Jianhui Yan, Wei Su

**Affiliations:** Department of Pediatrics, Affiliated Hospital of Xiangnan University, Chenzhou, 423000, Hunan, China; Clinical College of Xiangnan University, Affiliated Hospital of Xiangnan University, Chenzhou, 423000, Hunan, China; Department of General Practice, Affiliated Hospital of Xiangnan University, Chenzhou, 423000, Hunan, China

**Keywords:** ferroptosis, hypoxic-ischemic encephalopathy, neuroinflammation, carthamin yellow

## Abstract

Hypoxic–ischemic encephalopathy (HIE) is a common neurological disorder characterized by ischemia and hypoxia in the perinatal period, which seriously affects the growth and development of newborns. To date, there is no specific drug for the treatment of HIE. Previous studies have shown that ferroptosis plays an important role in the pathogenesis of HIE. Carthamin yellow (CY) is believed to have antioxidant and anti-inflammatory effects. However, no studies have reported the role of CY in ferroptosis in HIE *in vivo* until now. The aim of this study was to investigate the effect and mechanism of CY on HIE *in vivo* and to provide an experimental basis for the clinical treatment of HIE. The results demonstrated that CY increased the expression of NeuN in the neonatal rat hypoxic-ischemic brain damage (HIBD) model. Further exploration revealed that CY increased the expression of glutathione peroxidase 4 and ferritin heavy chain 1 while it decreased the expression of PTGS2 and ACSL2. Moreover, CY decreased malondialdehyde expression and increased superoxide dismutase and glutathione expression *in vivo*. The findings also indicated that CY downregulated the expression of Nrf2 and Keap-1. In conclusion, this study demonstrated that CY attenuated brain injury in an experimental HIBD model, potentially by alleviating hippocampal neuronal ferroptosis through inhibition of the Nrf2/Keap-1 signaling pathway. These findings provide a novel therapeutic strategy for the clinical treatment of HIE.

## 1 Introduction

Neonatal hypoxic–ischemic encephalopathy (HIE) is a complex perinatal condition caused by partial or complete hypoxia. This condition reduces or suspends cerebral blood flow and causes secondary hypoxic–ischemic brain damage (HIBD) [1]. HIE is also an important factor in neonatal mortality and prognostic impairment of mental development. Studies showed [2] that about 4 million newborns worldwide suffer from birth asphyxia each year, and about 25% of them develop HIE. This places a substantial socioeconomic burden on the society.

Ferroptosis is a new type of cell death discovered in recent years, caused by the iron-dependent accumulation of lipid-based reactive oxygen species (ROS). This process is regulated by the inactivation of glutathione peroxidase 4 (GPX4), which reduces lipid peroxidation at the expense of glutathione (GSH) [3]. The development of ferroptosis involves the participation of various proteins, such as acyl‑CoA synthetase long‑chain family member 4 (ACSL4), transferrin receptor 1, and ferritin heavy chain 1 (FTH1) [4,5]. ACSL4, an enzyme involved in lipid metabolism, plays a crucial role in iron accumulation, resulting in lipid peroxidation and iron overload [6]. FTH1, a prominent iron storage protein, is responsible for maintaining intracellular iron homeostasis [7]. Most importantly, it has been shown that ferroptosis is associated with ischemic stroke [8,9]. Increased levels of ROS and iron have been observed in the brains of ischemic stroke models [10].

Carthamin yellow (CY) is a flavonoid compound isolated from safflower. In Chinese medicine, CY is believed to enhance blood circulation and alleviate pain. As a result, it is commonly used in China for the treatment of coronary heart disease, cerebrovascular disease, and vasculitis [11]. Studies have demonstrated that CY possesses antioxidant and anti-inflammatory effects. Moreover, CY can inhibit lipopolysaccharide-induced activation of tumor necrosis factor-α [12]. Furthermore, other studies have shown that CY can exert a protective effect against myocardial ischemia-reperfusion injury by reducing the release of ROS and suppressing the inflammatory response [11]. Most importantly, CY has been also shown to ameliorate brain injury and improve neurological function in a rat model of middle cerebral artery occlusion by attenuating ferroptosis and neuroinflammation [13].

The above studies indicate that CY may have a potential therapeutic effect on ischemic diseases, but its mechanisms have not been fully elucidated and may be closely related to ferroptosis. However, there is no existing research on the effectiveness of CY in HIE and its relationship with ferroptosis. Therefore, this study utilized neonatal rats to investigate the impact of CY on HIBD models and to explore its potential molecular mechanisms. This research aims to offer a new therapeutic strategy and experimental foundation for the clinical treatment of HIE.

## 2 Materials and methods

### 2.1 Animals

SPF-grade mother rats and neonatal Sprague–Dawley rats on postnatal day 7 (P7) were obtained from the Charles River Laboratories. The neonatal Sprague–Dawley rats were randomly divided into the Sham group, HIBD + vehicle group, and HIBD + CY group. All rats were housed in an air-conditioned room with a 12 h light/dark cycle and were provided with adequate food and water. Efforts were made to reduce the number of animals used and to minimize rats suffering.

### 2.2 The HIBD model and CY administration

The neonatal rat HIBD model was established based on the study conducted by Rice et al. and Gou et al. [14,15]. Postnatal day 7 (P7) rats were anesthetized with isoflurane (induction concentration: 4%, maintenance concentration: 2%), and then the left common carotid artery was exposed and incised through the middle after double ligation with 6-0 sutures. Afterward, the rats were allowed to recover in a constant-temperature dam (37.0°C) for half an hour. Subsequently, the rats were placed in a hypoxic chamber (8% O_2_ + 9.2% N_2_ mixture (1.5 L/min)) for 2.5 h. In the Sham group, the common carotid artery exposure was without ligation or hypoxia. CY was obtained from Sigma Aldrich (Merck KGaA). CY was administered intraperitoneally to neonatal rats once daily for 2 weeks.

### 2.3 Behavioral test

In this study, we used the wire hang test for behavioral tests with reference to Chang et al. and Oliván et al. [16,17] on postnatal day 21. Briefly, a wire mesh grid (15 cm × 25 cm) was used. The rats were placed on the wire mesh grid 40 cm above a foam cushion. Then, the mesh was inverted 180°, and the rats were forced to grasp the wire using their four limbs. The hanging time was recorded as the duration the rats remained hanging before falling on the cushion. This test was repeated 5 times with an interval of 3 min between trials.

### 2.4 Immunofluorescence staining

Paraffin sections were dewaxed to water, and tissue sections were placed in a repair box filled with the EDTA antigen repair buffer for antigen repair in a microwave oven, medium heat for 8 min to boiling, cease-fire for 8 min, and then turned to medium-low heat for 7 min and cooled naturally. Then, the slides were placed in PBS (5 min × 3 times). BSA (3%) was used for sealing; the sealing liquid was thrown off and anti-NeuN (Abcam, ab177487, 1:200) was applied. The sections were incubated overnight at 4°C in a wet box, washed with PBS (5 min × 3 times), dried, and dropped with FITC-conjugated goat anti-rabbit IgG (1:200, Thermo Fisher, America) to cover the tissues, and incubated for 50 min at room temperature with DAPI at room temperature for 10 min with PBS, and then washed with PBS for 5 minand with water for 10 min. The slices were washed with PBS (5 min × 3 times) and then an autofluorescence quencher was added for 5 min, rinsed with running water for 10 min, and sealed with an anti-fluorescence quenching sealer. The slices were observed under a fluorescence microscope (BX53, OLYMPUS, Japan) under the same conditions.

### 2.5 Western blotting

Neonatal rat hippocampal tissues were extracted and total protein was obtained using RIPA buffer containing PMSF. The protein concentration was determined using the BCA assay kit (Beyotime, China) on postnatal day 21. Subsequently, the protein loading buffer was added and the samples were denatured at 95°C for 10 min. After that, the proteins were isolated by 10% SDS-polyacrylamide gel electrophoresis and then transferred to an activated PVDF membrane. Following blocking, the membrane was incubated overnight at 4°C with the appropriate concentration of primary antibody. Subsequently, the fluorescently labeled secondary antibody (IRDye700 and IRDye800, goat anti-rabbit) was incubated for 1 h at 37°C. Color rendering was performed using a chemiluminometer (Licor, America). The primary antibodies used were as follows: anti-NeuN (ab177487, abcam), anti-GPX4 (ab125066, abcam), anti-FTH1 (ybs-5907R, YOYOBIO), anti-PTGS2 (ab226869, abcam), anti-ACSL4 (PA5-27137, Invitrogen), anti-Nrf2 (PA5-27882, Invitrogen), and anti-Keap-1(ab227828, abcam).

### 2.6 Quantitative real-time polymerase chain reaction (qRT-PCR)

RNA was extracted and cDNA was synthesized following the instructions of the RevertAid First Strand cDNA Synthesis Kit (Thermo Fisher Scientific, America) on postnatal day 21. Real-time PCR was performed on the Applied Biosystems 7300/7500 real-time quantitative PCR instrument (Applied Biosystems, Thermo Fisher Scientific, America). GAPDH was used as an internal control. The primer sequence is shown in Table 1.

**Table 1:** Primer sequences for qRT-PCR

Gene	Forward primer (5′–3′)	Reverse primer (5′–3′)
GPX4	CTGCTCTTCCAGAGGTCCTG	GCCGTGTAGATATGGTACAAGGA
FTH1	AGGATATAAAGAAACCAGACCGTG	TCAGTAGCCAGTTTGTGCAG
PTGS2	GAGGGATCTGTGGATGCTTCG	AAACCCACAGTGCTTGACAC
ACSL4	TCCAAGCCAGAAAACTCAAGC	GGTGTACATGACAATGGCCAT
Nrf2	GTGGTTTAGGGCAGAAGG	TCTTTCTTACTCTGCCTCTA
Keap-1	GCTGTCCTCAATCGTCTCCT	ATTCGCCACTCGTTCCTCT
GAPDH	GAGTCAACGGATTTGGTCGT	GACAAGCTTCCCGTTCTCAG

### 2.7 ELISA

Hippocampus tissues were homogenized in PBS, and the supernatant was collected after centrifugation on postnatal day 21. GPX4 (No. JK-E4590; Shanghai Jing Anti-Bio Co., Ltd.; Shanghai, China), FTH1 (No. CSB-EL009030MO; Huamei Bio; Wuhan, China), PTGS2 (ZY-PTGS2-Mu; Shanghai ZeYe Biotechnology Co., Ltd; Shanghai, China), ACSL4 (HB-P9S2303X; Shanghai Chemical Biotechnology Co., Ltd; Shanghai, China), GSH (Item No. E-EL-0026c; Elab Biotechnology Co., Ltd; Wuhan, China), and rat ELISA kits were used according to the guidelines of the kits. Absorbance was measured by an enzyme marker (Bio-Rad). The levels of markers were determined from the standard curve and the levels were expressed as the calculated concentration/total protein.

### 2.8 Detection of malondialdehyde (MDA) and superoxide dismutase (SOD)

To assess the oxidative stress, hippocampal samples were collected and examined for SOD activity and MDA concentration. SOD activity was measured using the SOD (HP-E10001, HEPENGBIO) assay kit, and MDA concentration was determined using the MDA assay kit (S0131, Beyotime Biotechnology). The analysis was conducted on postnatal day 21.

### 2.9 Measurement of iron concentrations

Iron concentrations of hippocampal tissues and serum were measured using an atomic absorption spectrometer (model 180–80; Hitachi; Japan) after nitric acid digestion on postnatal day 21.

### 2.10 Statistical analysis

Statistical analysis was performed using Prism software (GraphPad 9.0, San Diego, CA, USA) and SPSS 26.0 (IBM, USA). All data were expressed as mean ± SD. The normality of the distribution was evaluated using the Kolmogorov–Smirnov test, and statistical analyses were conducted using one-way ANOVA with Tukey’s multiple comparison test used for the statistical analyses. *P* < 0.05 was considered statistically significant.


**Ethical approval:** The research related to animals’ use complied with all the relevant national regulations and institutional policies for the care and use of animals. All experimental procedures and animal care were approved by the Laboratory Animal Ethics Committee of Xiangnan University and were performed in accordance with the National Institutes of Health guidelines for the care and use of animals (Ethical approval number: no. 21C1786).

## 3 Results

### 3.1 CY ameliorates behavioral deficits and reduces hippocampal neuronal death in a neonatal rat HIBD model

**Figure 1:**
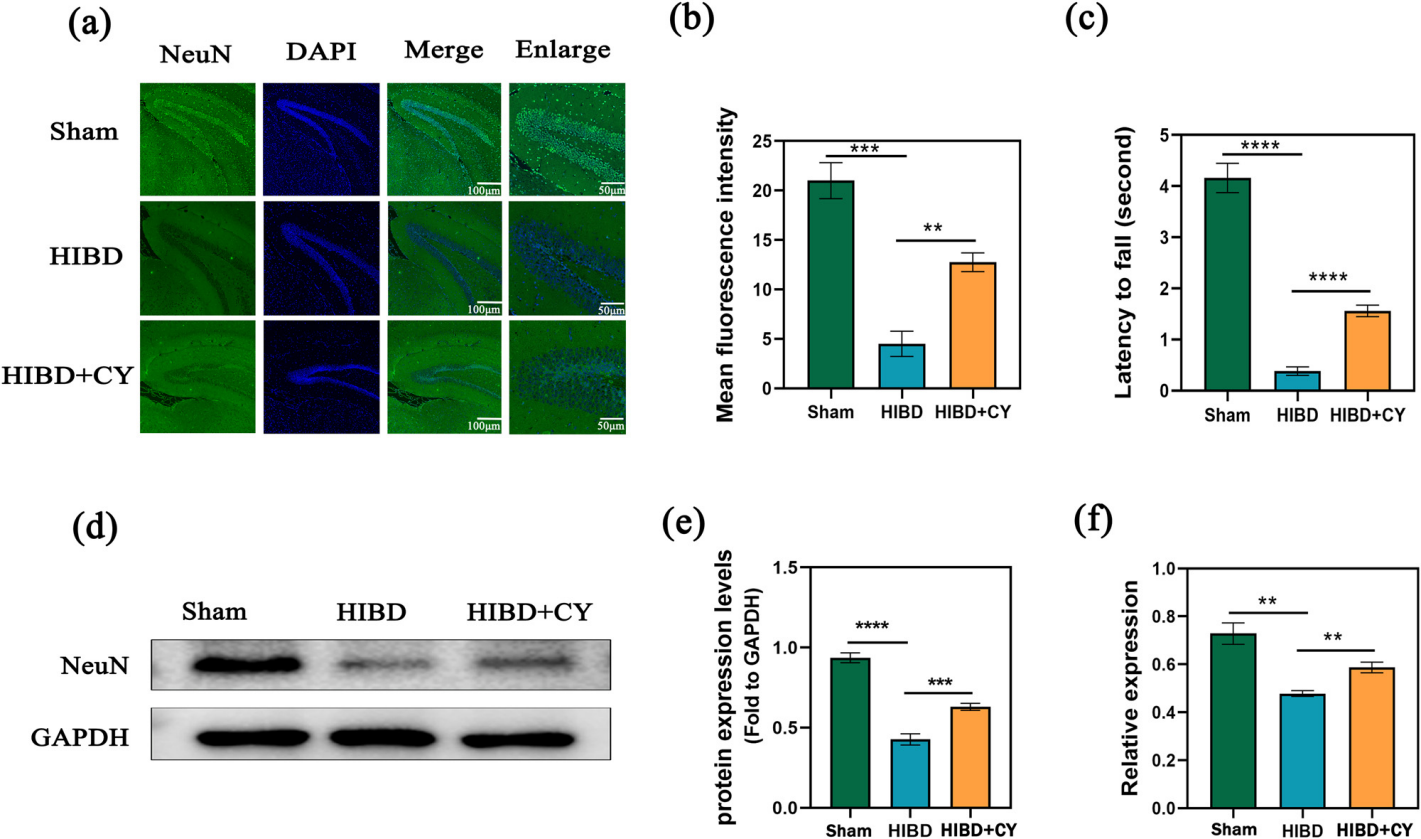
CY reduces hippocampal neuronal death in a neonatal rat HIBD model. (a) Immunofluorescence of NeuN in the hippocampus of a HIBD model; scale bar: 100, 50 μm. (b) Mean fluorescence intensity of NeuN in the hippocampus of a HIBD model. (c) Behavioral test of the HIBD model in neonatal rats. (d) Western blot of NeuN in the hippocampus. (e) Expression statistics of the NeuN protein (WB). (f) Relative mRNA expression of NeuN (qRT-PCR) **P < 0.01 and *** *P* < 0.001, *n* = 5.

To investigate the therapeutic effect of CY on the HIBD model, we performed a wire hang test on neonatal rats after CY treatment (on postnatal day 21). Our results demonstrated that the HIBD + vehicle group exhibited significant behavioral deficits compared with the Sham group. However, these deficits were significantly improved when CY (40 mg/kg) was administered (Figure 1c). Moreover, we examined the effect of CY on hippocampal neurons in the neonatal rat HIBD model by staining with hippocampal NeuN with immunofluorescence. We observed that the mean fluorescence intensity of the hippocampus in the HIBD + vehicle group was significantly lower than that in the Sham group, and the number of hippocampal neurons was effectively improved after CY (40 mg/kg) treatment (Figure 1a and b). Furthermore, we analyzed the protein expression of NeuN in the hippocampal tissue. The results indicated that the expression of NeuN was decreased in the HIBD + vehicle group compared with the Sham group, whereas the expression of NeuN was significantly increased in the HIBD + CY group compared with the HIBD + vehicle group (Figure 1d–f). These findings suggest that CY can effectively ameliorate behavioral deficits and reduce hippocampal neuronal death in the neonatal rat HIBD model.

### 3.2 CY inhibits ferroptosis in hippocampal neurons of the neonatal rat HIBD model

**Figure 2:**
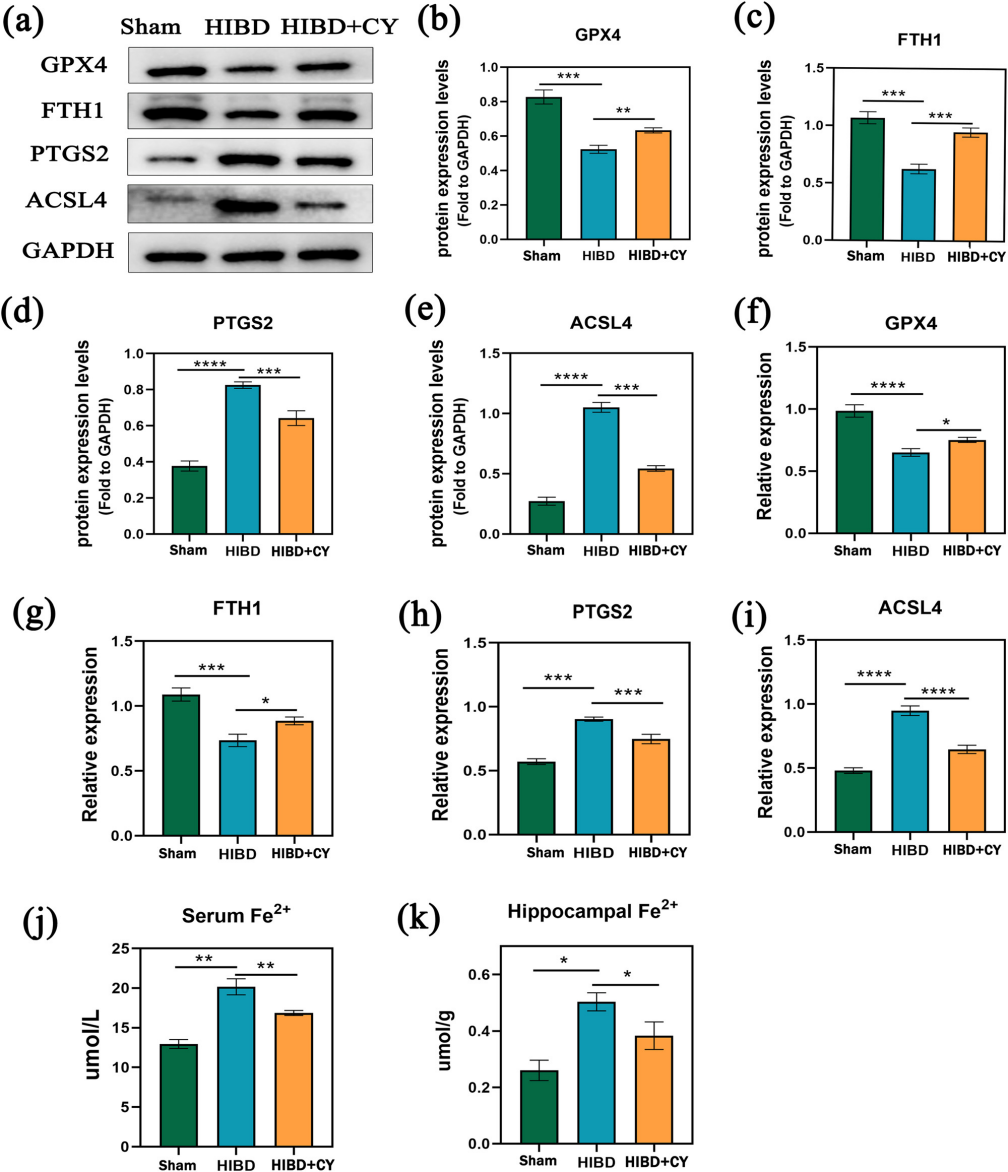
CY inhibits ferroptosis in hippocampal neurons of the neonatal rat HIBD model. (a) Western blot of GPTX4, FTH1, PTGS2, and ACSL4. (b) Expression statistics of the GPTX4 protein (WB). (c) Expression statistics of the FTH1 protein (WB). (d) Expression statistics of the PTGS2 protein (WB). (e) Expression statistics of the ACSL4 protein (WB). (f) Relative mRNA expression of GPX4 (qRT-PCR). (g) Relative mRNA expression of FTH1 (qRT-PCR). (h) Relative mRNA expression of PTGS2 (qRT-PCR). (i) Relative mRNA expression of ACSL4 (qRT-PCR). (j) Fe^2+^ content in serum. (k) Fe^2+^ content in the hippocampus. *P < 0.05; **P < 0.01; and ***P < 0.001, *n* = 5.

To explore the specific mechanism of CY on hippocampal neuronal death *in vivo*, we administered CY (40 mg/kg) to HIBD rats. Subsequently, we conducted a semi-quantitative analysis to detect GPX4, FTH1, PTGS2, and ACSL4. Also, we detected Fe^2+^ in hippocampal tissues, as well. The results showed that GPX4 and FTH1 were significantly decreased and PTGS2 and ACSL4 were significantly increased in the HIBD + vehicle group compared with the Sham group. In contrast, the expression of GPX4 and FTH1 was significantly increased and the expression of PTGS2 and ACSL4 was significantly decreased in the HIBD + CY group compared with the HIBD + vehicle group (Figure 2a–i). In addition, our results of Fe^2+^ detection in the hippocampal tissue and serum of the HIBD model suggested that Fe^2+^ was significantly increased in the HIBD + vehicle group compared with the Sham group. The Fe^2+^ was significantly decreased after the treatment with CY (Figure 2j and k). These results suggest that CY has the effect of inhibiting the process of ferroptosis in hippocampal neurons.

### 3.3 CY suppresses lipid peroxidation and improves antioxidant capacity

**Figure 3:**
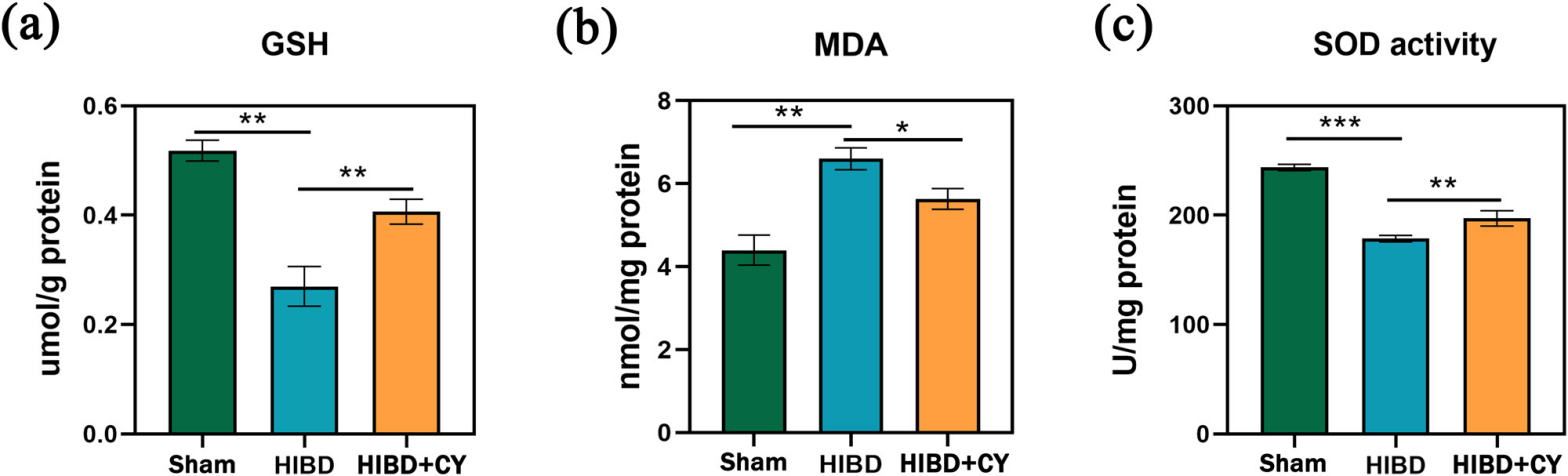
CY suppresses lipid peroxidation and improves antioxidant capacity. (a) GSH content in the hippocampus. (b) MDA content in the hippocampus. (c) SOD content in the hippocampus. *P < 0.05; **P < 0.01; and ***P < 0.001, *n* = 5.

In order to further investigate the effect of CY on lipid peroxidation capacity and antioxidant capacity in the HIBD model, we examined the expression levels of MDA, SOD, and GSH in hippocampal tissues. The results showed that compared with the HIBD + vehicle group, the HIBD + CY group effectively reduced the expression of MDA in the neonatal rat HIBD model (Figure 3b). In addition, CY also effectively improved the expression of SOD and GSH (Figure 3a and c). These results indicate that CY treatment can effectively inhibit the lipid peroxidation process and improve the antioxidant capacity in the neonatal rat HIBD model.

### 3.4 CY represses the activation of the Nrf2/Keap-1 signaling pathway

**Figure 4:**
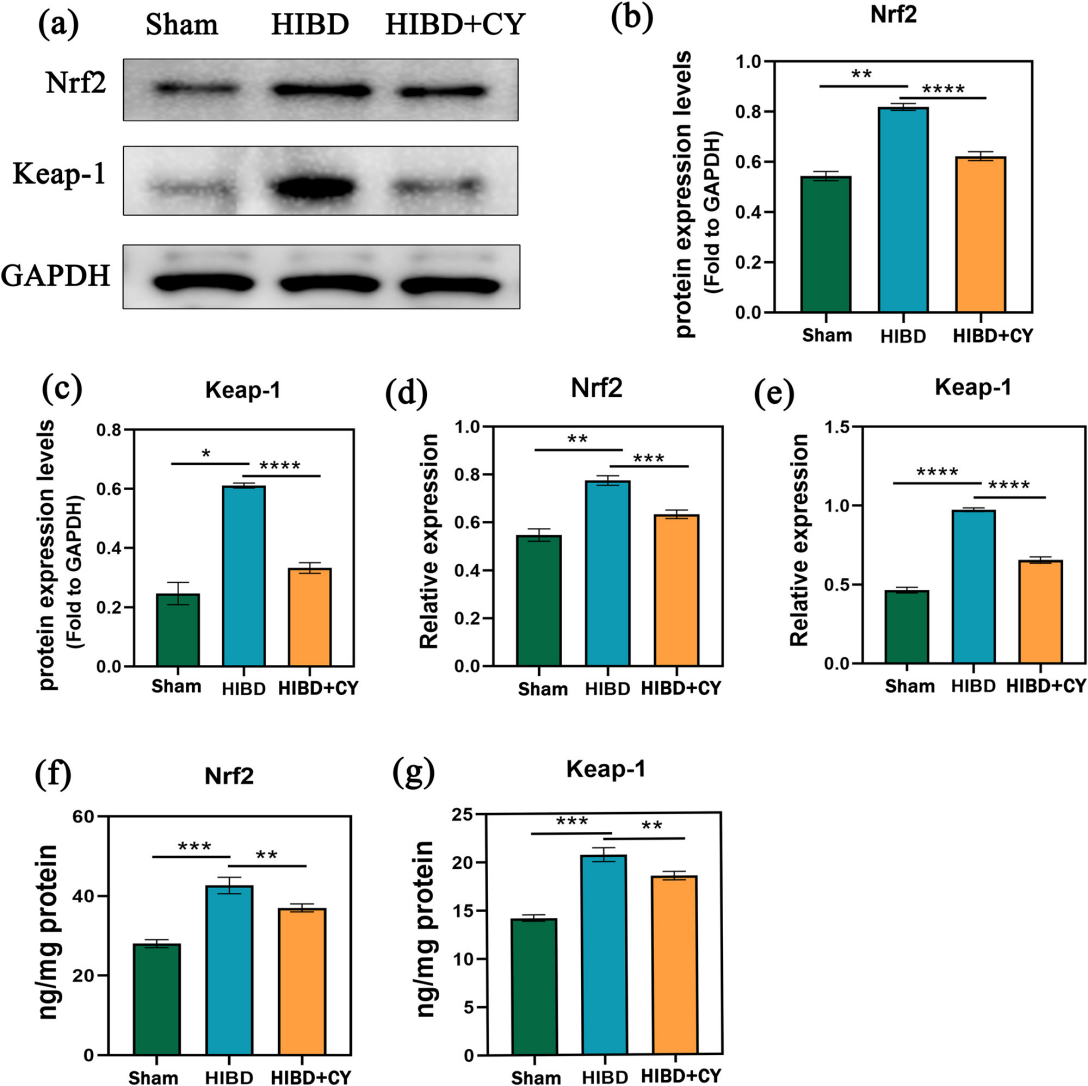
CY represses Nrf2/Keap-1 signaling pathway activation. (a) Western blots of Nrf2 and Keap-1. (b) Expression statistics of Nrf2 protein (WB). (c) Expression statistics of the Keap-1 protein (WB). (d) Relative mRNA expression of Nrf2 (qRT-PCR). (e) Relative mRNA expression of Keap-1 (qRT-PCR). (f) Expression of Nrf2 in the hippocampus (ELISA). (g) Expression of Keap-1 in the hippocampus (ELISA). *P < 0.05; **P < 0.01; and *** *P* < 0.001, *n* = 5.

To investigate the deeper mechanism of CY in attenuating ferroptosis, we examined the relative mRNA levels and protein expression levels of Nrf2 and Keap-1. We observed that the relative mRNA expression and protein expression levels of Nrf2 and Keap-1 were higher in the HIBD + vehicle group compared with the Sham group. When CY treatment was given, the relative mRNA expression and protein expression levels of Nrf2 and Keap-1 decreased (Figure 4a–g). These findings suggest that CY not only affects the protein expression of Nrf2 and Keap-1 but also influences their gene expression.

## 4 Discussion

Neonatal HIE causes neonatal hypoxic–ischemic brain injury is a devastating neurological injury that results from a decrease in the oxygen and blood flow to the brain during birth. Currently, the available treatment modalities are more limited, and thus there is still a high morbidity and mortality rate after neonatal HIE. Hence, it is crucial to explore new therapeutic strategies. Ferroptosis is a relatively new mechanism of cell death that differs from other types of cell death, such as apoptosis, necrosis, and pyroptosis [18,19]. Ferroptosis and ischemic brain injury have been shown to be associated [8,9]. Although CY exhibits various biological activities including antioxidant and anti-inflammatory properties, its impact on HIBD models has not been reported. In this study, we report for the first time that CY attenuates brain injury in a HIBD model and achieves this effect by inhibiting ferroptosis in the hippocampal neurons of rats.

In this study, we first investigated the effect of CY on hippocampal neurons in a neonatal rat HIBD model. Our findings demonstrate that CY reduces hippocampal neuronal death, providing the first evidence of its positive therapeutic effect *in vivo*. In addition, to explore the death pattern of hippocampal neurons in the neonatal rat HIBD model, we explored core markers of ferroptosis, GPX4, FTH1, PTGS2, and ACSL4. Our study found that the expression of GPX4 and FTH1 was significantly down-regulated and PTGS2 and ACSL4 were significantly up-regulated in the hippocampal tissue of the neonatal rat HIBD model, which represents the occurrence of ferroptosis in hippocampal neurons in the neonatal rat HIBD model. These findings are consistent with the studies by Lin et al. and Zhu et al. [20,21]. However, it is noteworthy that neither of them explored the effect and mechanism of CY on HIBD. In our study, we found that CY could effectively upregulate the expression of GPX4 and FTH1 and downregulate the expression of PTGS2 and ACSL4. This indicates that CY can effectively alleviate ferroptosis in hippocampal neurons of neonatal rat HIBD models. Although CY has been shown to attenuate ferroptosis in an adult rat model of ischemic brain injury, therapeutic strategies for neonatal ischemic injury are relatively lacking due to the different pathogenesis of adult brain ischemic injury and neonatal ischemic injury. Therefore, our study provides important insights by demonstrating the effectiveness of CY in ameliorating hippocampal neuronal ferroptosis in a neonatal rat model of HIBD *in vivo*, which offers a valuable addition to the therapeutic options for neonatal ischemic–hypoxic injury. Moreover, we investigated the effect of CY on the expression of Fe^2+^ in the neonatal rat model of HIBD by examining the Fe^2+^ levels in the hippocampal tissue. Our results showed that CY effectively reduced the Fe^2+^ levels in the hippocampal tissue. This is consistent with the findings of Guo et al. [13].

Furthermore, we also investigated the effects of CY on lipid peroxidation and antioxidation. We found that CY can inhibit lipid peroxidation and improve antioxidant capacity. This is similar to the results of Guo et al. and Zhu et al. [13,21]. However, it is worth noting that their research did not specifically investigate the effect of CY on the neonatal rat HIBD model *in vivo*. These results further suggest that CY can reduce ferroptosis in hippocampal neurons.

In order to further explore the mechanism of CY in attenuating ferroptosis, we explored the relevant signal pathways at the genetic and molecular levels. We found that the expression of Keap-1 and Nrf2 was significantly up-regulated in the neonatal rat HIBD model, while the expression of Keap-1 and Nrf2 decreased when treated with CY, which is observed for the first time in the neonatal rat HIBD model. Recent reports have confirmed that the target gene of Nrf2 plays an important role in ferroptosis, including GPX4, GSH, FPN1, and so on [22]. Nrf2 plays a positive role in the regulation of ferroptosis, that is, the sensitivity of ferroptosis is positively correlated with the expression level of Nrf2 [22]. Under normal organismal conditions, Kelch-like ECH-associated protein 1 (Keap-1) maintains Nrf2 function by regulating proteasomal degradation and ubiquitination of Nrf2; however, when oxidative stress is activated, disulfide bonds are formed between Cys288 and Cys273 in Keap-1, resulting in detachment and translocation of Nrf2, which translocates to the nucleus by binding to Nrf2 translocated to the nucleus, and can translocate downstream SLC7A11 to the cell membrane by binding to antioxidant response elements and coupling to SLC3A2, and overexpression of Keap-1 can disrupt these processes and thus trigger ferroptosis [23]. In a recent study by Li et al. [24], it was shown that neurological injury is closely associated with ferroptosis, and the p62-Keap-1-Nrf2 pathway after arterial hemorrhage can reduce ferroptosis by mediating downstream ferroptosis markers to attenuate neurological injury in arterial hemorrhage patients. Our findings are similar to this, and we demonstrate that CY inhibits the Nrf2/Keap-1 signaling pathway. This suggests that the mechanism by which CY inhibits ferroptosis in hippocampal neurons may be related to the Nrf2/Keap-1 signaling pathway.

In this study, we first found that CY could inhibit hippocampal neuronal death in HIBD rats, and further demonstrated that CY inhibited hippocampal neuronal death by ferroptosis. Additionally, we also confirmed that CY can suppress the activation of the Nrf2/Keap-1 signal pathway, which is closely related to ferroptosis. In summary, our study suggests that CY has the potential to be a therapeutic drug for the clinical treatment of HIE in the future, which is worth further development and research. Although we demonstrated that CY attenuates brain injury in the HIBD model, there are still some limitations of this study. First, we focused solely on investigating the effects of CY *in vivo* and did not explore its impact at the cellular level. Second, we did not set a concentration gradient of CY in this study as the effective concentration of CY has been previously reported in the literature. Third, due to the limitations of conditions, we were unable to measure the infarct area of HIBD using TTC staining. Therefore, future studies should aim to address these limitations and refine these experiments to enhance the level of evidence.

## 5 Conclusion

CY significantly attenuated brain injury in an experimental neonatal rat HIBD model and the mechanism may be through the inhibition of the Nrf2/Keap-1 signaling pathway to attenuate hippocampal neuronal ferroptosis. Furthermore, our findings provide a novel therapeutic strategy for HIE.
